# Interdependency of regulatory effects of iron and riboflavin in the foodborne pathogen *Shigella flexneri* determined by integral transcriptomics

**DOI:** 10.7717/peerj.9553

**Published:** 2020-09-15

**Authors:** Luis Fernando Lozano Aguirre, Juan Carlos Salazar, José Ignacio Vásquez, Víctor Antonio García-Angulo

**Affiliations:** 1 Unidad de Análisis Bioinformáticos, Centro de Ciencias Genómicas, Universidad Nacional Autónoma de México, Cuernavaca, Morelos, México; 2 Programa de Microbiología y Micología, Instituto de Ciencias Biomédicas, Universidad de Chile, Santiago, Chile; 3 Department of Ocean Sciences, Memorial University of Newfoundland, St. John, Canada

**Keywords:** Transcriptomics, Iron, Riboflavin, *Shigella flexneri*, Regulation

## Abstract

*Shigella flexneri* is the causative agent of dysentery. For pathogens, iron is a critical micronutrient as its bioavailability is usually low in bacterial niches. This metal is involved in critical physiological processes mainly as a component of important metabolic molecules involved in redox reactions. Usually bacteria respond to fluctuations in iron availability to regulate iron acquisition and other iron-related functions. Recently the close metabolic feedback between iron and riboflavin, another pivotal biological redox agent, began to draw attention in bacteria. This is a widespread biological phenomenon, partly characterized by the coordination of regulatory responses to iron and riboflavin, probably owed to the involvement of these cofactors in common processes. Nonetheless, no systematic analyses to determine the extent of this regulatory effect have been performed in any species. Here, the transcriptomics responses to iron, riboflavin, iron in the presence of riboflavin and riboflavin in the presence of iron were assessed and compared in *S. flexneri*. The riboflavin regulon had a 43% overlap with the iron regulon. Notably, the presence of riboflavin highly increased the number of iron-responsive genes. Reciprocally, iron drastically changed the pool of riboflavin-responsive genes. Gene ontology (GO) functional terms enrichment analysis showed that biological processes were distinctively enriched for each subgroup of responsive genes. Among the biological processes regulated by iron and riboflavin were iron uptake, amino acids metabolism and electron transfer for ATP synthesis. Thus, iron and riboflavin highly affect the transcriptomics responses induced by each other in *S. flexneri*. GO terms analysis suggests that iron and riboflavin coordinately regulate specific physiological functions involving redox metabolism.

## Introduction

*Shigella flexneri* is one of four known *Shigella* species causing dysentery. The disease is clinically characterized by acute watery diarrhea, which sometimes includes bleeding, fever and abdominal cramps (Mattock & Blocker, 2017). In rare cases, complications of the disease include haemolytic-uremic syndrome and post-reactive arthritis. *Shigella* is restricted to the human host and invades enterocytes, surviving freely in the cytoplasm after lysis of the macropinocytic vacuole through which it enters the cells. As for other pathogenic bacteria, iron is a critical micronutrient for *S. flexneri*. Although this is one of the most common elements on earth, biologically available forms of iron are present at extremely low levels in many bacterial niches due to the oxidative conditions of the environment and to sequestration by iron-binding proteins and compounds within hosts (Wei & Murphy, 2016; Sheldon, Laakso & Heinrichs, 2016; Begg, 2019). With a few documented exceptions, iron is essential for bacterial species mainly as an electron transfer enzymatic cofactor. Transitions between ferric (Fe^3+^) and ferrous (Fe^2+^) states in the active sites of metalloproteins facilitate redox reactions that are required during critical biological processes such as respiration, oxidative stress response, activation of metabolites and nucleic acids biosynthesis among many others (Sánchez et al., 2017; Begg, 2019).

Bacteria have developed several pathways to obtain iron. *S. flexneri* can uptake ferric iron through the siderophore aerobactin, which is synthesized and internalized by the products of the *iucABCD*, *iutA* and *fhu* genes. This species can also internalize ferric dicitrate by the proteins encoded in the *fecABCDE* locus (Vokes et al., 1999; Luck et al., 2001; Wei & Murphy, 2016). Ferrous iron can be acquired by *S. flexneri* using the Feo and the Sit transporter systems encoded by the *feoABC* and *sitABCD* loci (Runyen-Janecky et al., 2003; Wei & Murphy, 2016). The variation in concentration and types of iron species within niches has important effects on the bacterial physiological status. Iron uptake and other functions related to iron homeostasis are tightly regulated to maintain an equilibrium between iron supply and demand, as an excess of iron is detrimental due to generation of reactive oxygen species (Sheldon, Laakso & Heinrichs, 2016). Iron regulatory effects in bacteria are achieved mainly through the transcriptional regulator Fur, which dimerizes and binds DNA regulatory sequences upon interaction with intracellular iron. In addition, examples of Fur-independent iron regulatory effects exist (Carpenter, Whitmire & Merrell, 2009; Wei & Murphy, 2016; Sheldon, Laakso & Heinrichs, 2016). Given the importance of this micronutrient, many studies have assessed the global regulatory effects of iron on bacterial gene expression. In many cases, the genes coding for riboflavin biosynthesis and uptake systems are targets for iron regulation (Sepúlveda Cisternas, Salazar & García-Angulo, 2018). Riboflavin is a precursor for flavin mononucleotide and flavin adenine dinucleotide, which together with iron, comprise the most important redox cofactors across life kingdoms (Monteverde et al., 2016). Bacteria can biosynthesize riboflavin through the riboflavin biosynthetic pathway (RBP) composed by the RibA, RibB, RibD, RibH and RibE proteins and/or internalize it from the environment using flavin uptake systems (García-Angulo, 2016). *S. flexneri* encodes a full RBP but lacks any known riboflavin importers (Gutiérrez-Preciado et al., 2015). In some bacterial species, overexpression of RBP genes in response to a decrease in environmental iron has been described (Vasileva et al., 2012; Da Silva Neto, Lourenço & Marques, 2013; Nguyen et al., 2016; Berges et al., 2018; Jiménez-Munguía et al., 2018). Recently, our group has found evidence for the reciprocal phenomenon in the diarrheagenic pathogen *Vibrio cholerae*, where exogenous riboflavin regulates the expression of a gene required for the function of various iron uptake systems. In *V. cholerae*, the riboflavin regulon determined by transcriptomics includes many genes known to be regulated by iron (Sepúlveda-Cisternas et al., 2018). Thus, it seems that a widespread cross-regulatory effect of riboflavin and iron exists in bacteria. This may be the reflection of a largely known metabolic relationship between these two molecules functioning as central redox cofactors present both in prokaryotes and eukaryotes (Hickey, 1945; Dmytruk et al., 2014; Chen, Wang & Yeh, 2017; Sepúlveda Cisternas, Salazar & García-Angulo, 2018). It has been hypothesized that riboflavin overproduction may contribute to supporting growth during iron starvation conditions. Riboflavin may increase iron bioavailability by mediating ferrous iron reduction and by boosting hemolysis (Worst et al., 1998; Crossley et al., 2007). Flavins also work as cofactors for some components of iron uptake systems and thus their overproduction may improve iron internalization (Chai et al., 2015; Kobylarz et al., 2017). Finally, flavins can be cofactors of flavoenzymes that replace iron-requiring enzymes (Thamer et al., 2003; Chazarreta-Cifre et al., 2011). Although the iron-riboflavin regulatory overlay seems to be paramount in bacterial redox metabolism, no systematic approach has been applied to study the global feedback between iron and riboflavin during the regulation of gene expression. In order to characterize the global effects of iron in *S. flexneri*, this study performed a transcriptomics analysis to identify genes differentially expressed in response to iron in the medium. In addition, to gain a global insight into the regulatory relationship between iron and riboflavin in this species, transcriptomics responses to riboflavin and the reciprocal effects of iron and riboflavin in such transcriptomes were determined.

## Materials & Methods

### Growth conditions, RNA extraction and sequencing

*Shigella flexneri* strain 2457T (available at American type cell culture strain ATCC^®^700930™) was grown in trypticase soy agar (TSA) with Congo red (0.01%) at 37 °C overnight. Five colonies positive for *virF* amplification, and therefore positive for the presence of the virulence plasmid, were used to inoculate 5 ml of Lysogeny Broth (LB) and incubated at 37 °C and 180 rpm shake. Next, 1 ml of this culture was centrifuged at 12,000 rpm on a standard tabletop centrifuge and supernatant discarded. Pellet was washed three times with T minimal medium (Wyckoff & Payne, 2011) without iron and resuspended in 1 ml of the same medium. 500 µl of this inoculum were subcultured in flasks containing 25 ml of T medium. When indicated, T medium minus iron was supplemented with 40 µM FeCl_3_ and/or 2.5 µM riboflavin. These concentrations of iron and riboflavin have proven to induce transcriptomics changes in *V. cholerae* (Sepúlveda-Cisternas et al., 2018). Cultures were incubated at 37 °C with 180 rpm shaking until mid-log phase (approximately 12 h). At this time, optical density (O.D._600_) of cultures was near 0.4. Cultures without iron took approximately two more hours to reach similar O.D._600_ values. RNA was extracted with the Thermo Scientific Genejet RNA purification kit according to the manufacturer’s instructions. Residual DNA in the RNA preparations was eliminated using the Turbo DNA-free DNase kit for 1 h at 37 °C. RNA samples from three independent biological replicates for each condition were sequenced using the Illumina HiSeq platform to produce 100 bp paired-end reads, with ∼40 million reads per sample at Macrogen (Korea). RNA quality control results for each sample are indicated in the Table  S1.

### RNASeq data analysis

Quality analyses and trimming of RNASeq raw reads were performed with FastqQC v.0.11.2 (https://www.bioinformatics.babraham.ac.uk/projects/fastqc/) and Trim Galore v0.4.1 (http://www.bioinformatics.babraham.ac.uk/projects/trim_galore/), respectively. Bowtie2 v.2.3.4.2 (Langmead & Salzberg, 2012) was used to align the reads to the genome of *S. flexneri* strain 2457T. Differential expression analysis was performed with EdgeR v.3.18.1 from the Bioconductor package (Robinson, McCarthy & Smyth, 2010) with a negative binomial model. Genes with a statistically significant change in expression (*p* < 0.05) were selected for Gene Ontology Enrichment Analysis (Fisher’s exact test, corrected *p*-value by False Discovery Rate control and Filter value of 0.05) with Blast2GO (https://www.blast2go.com/).

## Results

**Figure 1:**
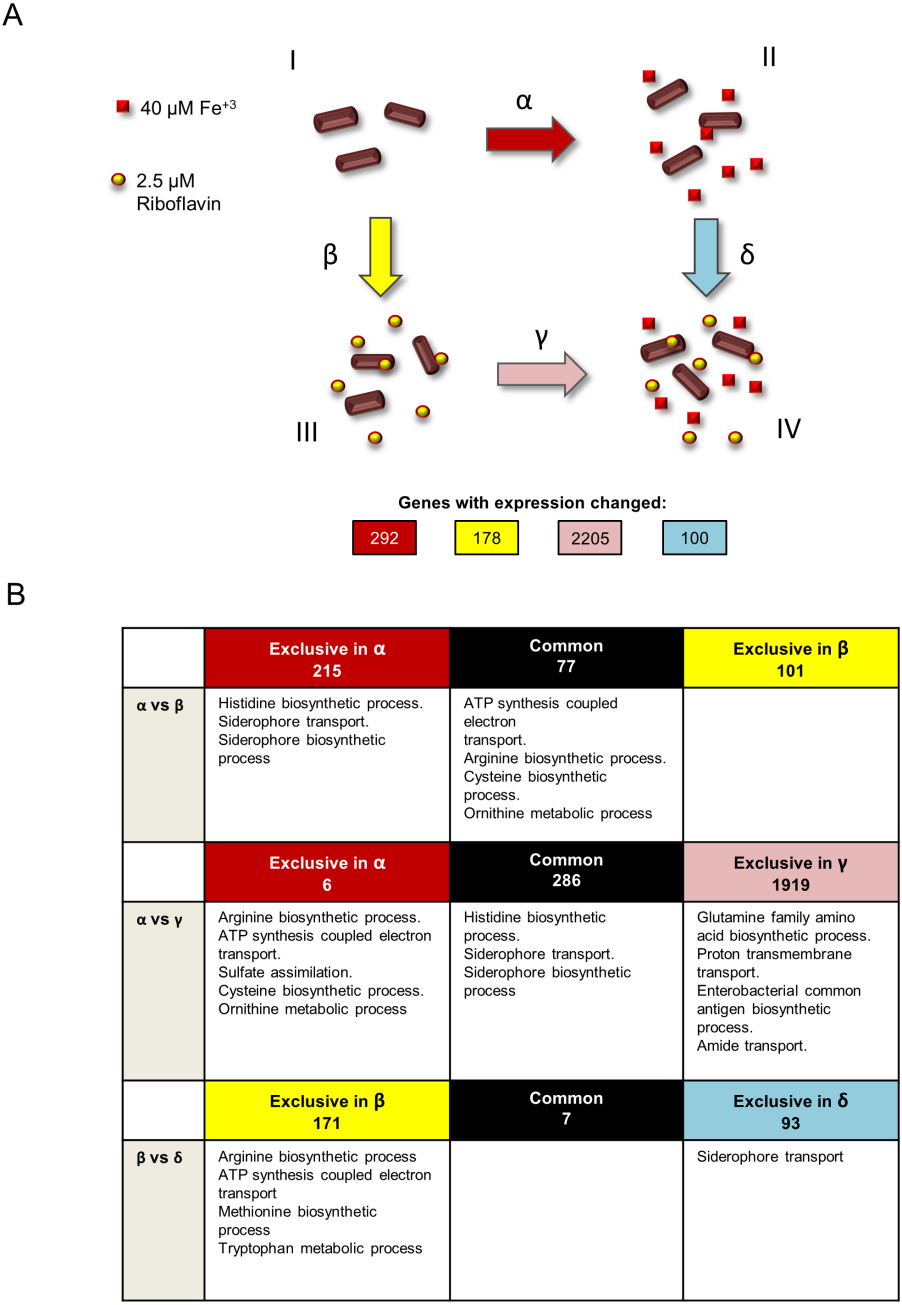
Experimental design and summary of results of transcriptomics comparisons. (A) Growth conditions and comparisons accomplished in the transcriptomics analysis performed in this study. This study included four independent *S. flexneri* growth conditions: I, T medium without iron. II, T medium with iron. III, T medium plus riboflavin; IV, T medium plus iron plus riboflavin. In order to determine genes whose expression is affected by iron, RNAseq was compared between bacteria grown in T media versus bacteria grown in T medium plus iron (comparison *α*). Comparison *β*, transcriptome of bacteria grown in T media versus transcriptome of bacteria grown in T medium plus riboflavin, identified genes affected by riboflavin. Comparison *γ*, bacteria grown in T medium plus riboflavin versus bacteria grown in T medium plus riboflavin plus iron, identified genes affected by iron in the presence of riboflavin. Conversely, comparison *δ*, bacteria grown in T medium plus iron versus bacteria grown in T medium plus iron plus riboflavin, identified genes affected by riboflavin when iron is present. Transcriptomics and statistical analysis were performed as described in Materials and methods. Three independent cultures and libraries constructions were performed for each growth condition. (B) Schematic representation of the comparison of transcriptomics effects of riboflavin and iron and their reciprocal effects, showing the number of genes regulated in each comparison performed and the GO terms statistically enriched on each subset of genes. Empty boxes indicate that no GO terms were enriched in the subset of genes.

*S. flexneri* was grown in four independent conditions: minimal T medium without iron (growth condition I), complete T medium with iron (growth condition II), T medium without iron plus riboflavin (growth condition III) and T medium plus iron and riboflavin (growth condition IV) (Fig. 1A). Total RNA was extracted from three independent cultures for each condition and sequenced using Illumina HiSeq. Four statistical comparisons were performed in order to identify genes with differential expression in the media conditions tested: *α*, T medium minus iron versus complete T medium plus iron (condition I versus II); *β*, T medium minus iron versus T medium minus iron plus riboflavin (condition I versus III); *γ*, T medium minus iron plus riboflavin versus complete T medium plus iron and riboflavin (condition III versus IV); and *δ*, complete T medium plus iron versus complete T medium plus iron and riboflavin (conditions II versus IV). The expression of 292, 178, 2205 and 100 genes was significantly changed (*P* < 0.05), respectively.

### Effect of iron in transcription in *S. flexneri*

In comparison *α* (Fig. 1A), 292 genes were responsive to iron. Of these, 99 genes were induced and 193 genes were repressed. Both categories included many genes known to be affected by iron in other bacteria (Table S2). For example, among the iron-induced genes was *sodB*, encoding an iron superoxide dismutase, a well know target for activation by the iron-Fur complex in *Escherichia coli* (Dubrac & Touati, 2000). Also, the *nuo* operon encoding a NADH dehydrogenase of the respiratory chain, as well as other energy metabolism genes, were upregulated by iron as reported previously in *E. coli* (McHugh et al., 2003). Notably, the *ftnA* gene, encoding a cytoplasmic ferritin involved in iron storage, was induced by the presence of iron, which is an expected effect of high iron availability. In addition, genes for the arginine, histidine and thiamine amino acids biosynthesis and for components of the 50S and 30S ribosomal subunits are included in this comparison. Twenty genes involved in iron acquisition were highly repressed by iron (Table S2). This result is in agreement with several transcriptomics analyses in response to iron previously reported in other bacteria (Mey et al., 2005; Da Silva Neto, Lourenço & Marques, 2013; Pajuelo et al., 2016; Kurthkoti et al., 2017; Berges et al., 2018). The *fes, fepABCDG*, *entDF*, *iutA, fhuAF* and *iucABCD* genes, all involved in siderophore biosynthesis and utilization, were repressed by iron. Of these, the most downregulated was *fes,* with a 21-fold change repression in the presence of iron. The degree of repression of the rest of these genes ranged from 2.5 (*fhuA*) to 19.7 (*fepA*) fold. The gene *exbD,* coding for a component of the TonB/ExbB/ExbD complex that energizes siderophore uptake, was also repressed by iron (1.77-fold change). The genes *feoA* and *sitABCD*, which encode components of the two different Fe^2+^ acquisition systems present in *S. flexneri*, were also repressed by iron, with changes ranging from 1.8 to 4.8 fold. In general, bacterial iron acquisition systems are mainly repressed under high iron conditions, and specifically the *feo* and *sit* genes have already been reported to be downregulated by iron in *S. flexneri* (Wyckoff, Boulette & Payne, 2009). Although often the expression of riboflavin biosynthetic genes is responsive to iron in bacteria (McHugh et al., 2003; Da Silva Neto, Lourenço & Marques, 2013; Berges et al., 2018), no such genes were differentially expressed in this comparison. Nonetheless, the open reading frame cds2502, coding for a putative flavodoxin, was repressed by iron (Table S2). Accordingly, growth under iron-scarcity conditions has been postulated to be supported by increased expression of flavodoxins to replace ferredoxins in other bacteria (Baichoo et al., 2002; Thamer et al., 2003; Nguyen et al., 2016; Berges et al., 2018). To better understand the global effects of the transcriptional changes induced by iron, an analysis of enrichment of Gene Ontology (GO) functional terms on the set of iron-responsive genes was performed. Overall, seven GO terms were found to be enriched in this group: *histidine biosynthetic process, siderophore transport, siderophore biosynthetic process,* which were downregulated, and *ATP synthesis coupled electron transport, arginine biosynthetic process*, *cysteine biosynthetic process* and *ornithine metabolic process*, which were upregulated*.*

### Transcriptomics responses to riboflavin

In the transcriptomics results, 178 genes responded to riboflavin. Of these, 48 were upregulated and 130 were repressed (Table S2). Among the induced genes, many encoded hypothetical, phage and insertion sequences proteins. Also, a putative oxidoreductase, a putative cytochrome c type protein and type 3-secreted protein genes were included in this list. Among repressed genes, we found some encoding flavoproteins, like the sulfite reductase CysJ and many energy metabolism and amino acid metabolic proteins. In a previous study, our group performed a transcriptomic analysis of the response to riboflavin in *V. cholerae* (Sepúlveda-Cisternas et al., 2018). Many genes that responded to riboflavin or to the elimination of riboflavin supply pathways in that study are also regulated by riboflavin in *S. flexneri*. For example, *sodB*, genes for ribosomal proteins and NADH dehydrogenase I component genes were repressed by riboflavin in *V. cholerae* and also in *S. flexneri*. Similarly, c-type cytochrome biogenesis genes were activated by riboflavin in both bacterial species.

Our preceding study showed that the *V. cholerae* riboflavin regulon includes many genes known to be regulated by iron. Likewise, riboflavin-responsive genes in *S. flexneri* in comparison *β* included many iron-responsive genes. In order to investigate the degree of overlap between the iron and riboflavin regulons in *S. flexneri*, genes affected by each were compared. This comparison showed that 77 out of 178 genes (roughly 43%) responding to riboflavin also responded to iron (Fig. 1B, first row, *α* versus *β*). Notably, in all cases riboflavin had the opposite effect of iron on the expression of these genes (Fig. S1). Among the genes responsive to both iron and riboflavin, four GO terms were enriched: *ATP synthesis coupled electron transport*, *arginine biosynthetic process, cysteine biosynthetic process* and *ornithine metabolic process*. Thus, these biological processes seem to be regulated in common by iron and riboflavin, but while iron induces them, riboflavin represses them. It is noteworthy that no iron uptake genes were found in this set of genes. Hence, the GO terms related to iron acquisition are enriched only in the set of genes exclusively regulated by iron. This suggests that the regulatory overlay effect observed between riboflavin and iron seems not to affect the activation of iron acquisition genes in low iron. In other words, the expression of iron uptake genes is high under a low iron condition, irrespective of the presence of riboflavin. Finally, no GO term was enriched among the 101 genes responsive to riboflavin but not to iron.

### Reciprocal effect of iron and riboflavin on their transcriptional outcomes

In *V. cholerae*, iron exerts a regulatory effect over the expression of iron uptake genes and also over riboflavin biosynthetic genes. This regulation is dependent on riboflavin. Reciprocally, riboflavin regulation of riboflavin biosynthesis and iron uptake genes depends on iron (Sepúlveda-Cisternas et al., 2018). To investigate a similar effect on *S. flexneri* at the global level, the iron regulon in the presence of riboflavin (comparison *γ*) was set side by side to the iron regulon in the absence of riboflavin (comparison *α*). Strikingly, the number of genes responsive to iron in the presence of riboflavin increased to 2,205 (1,042 induced, 1163 repressed, Table S2), compared to the 292 affected by iron without riboflavin (Fig. 1B, second row, *α* versus *γ*). All but six of these 292 genes were also responsive to iron in the absence of riboflavin. Functional enrichment analysis in the set of 2,205 genes indicated that in addition to the three GO terms *histidine biosynthetic process*, *siderophore transport* and *siderophore biosynthetic* process already found regulated by iron, four other biological processes were regulated by iron in the presence of riboflavin: *glutamine family amino acid biosynthetic process*, *proton transmembrane transport*, *enterobacterial common antigen biosynthetic process* and *amide transport*. Notably, the *ribB* and *ribH* riboflavin biosynthetic genes were among the genes induced by iron in this condition. In the subset of genes affected by iron specifically in the absence of riboflavin we found an enrichment of the functional terms *sulfate assimilation*, *ATP synthesis coupled electron transport* and the metabolism of the amino acids *cysteine* and *ornithine*. Overall, the results of this comparison indicate that the iron regulon is highly increased by the presence of external riboflavin.

To investigate the reciprocal response, the regulons of riboflavin with iron (comparison *δ*) and without iron (comparison *β*) were matched. A total of 100 genes changed expression in response to riboflavin when iron was present in the medium (67 induced, 33 downregulated). Remarkably, only 7 of them were also responsive to riboflavin when iron was lacking (Fig. 1B, third row, *β* versus *δ*). The *siderophore transport* GO term was enriched in the subset of genes specifically regulated by riboflavin in iron. This is also one of the biological processes regulated by iron in the absence of riboflavin. Overall, these results indicate that the riboflavin regulon is highly modified by external iron.

## Discussion

This study determined the transcriptional responses to iron and riboflavin and the extent of the interdependencies of such regulons for the first time in any bacteria. Bacteria are known to reprogram their metabolic status depending on the presence of iron in the environment. Similar to reports in many other species*, S. flexneri* iron uptake system genes and other iron-metabolism related genes were downregulated in the presence of iron. The biological functions *ATP synthesis coupled electron transport*, as well as *biosynthesis of arginine, cysteine* and *metabolism of ornithine* were induced by iron, while *histidine biosynthesis* and iron uptake-related functions were repressed. This is consistent with previous reports studying iron effects on bacteria by high throughput approaches. It has been shown that iron has a major outcome on the energetic state of bacteria. Iron depletion induces a general downregulation of oxidative phosphorylation and general respiratory complexes in *E. coli* (McHugh et al., 2003), *V. cholerae* (Mey et al., 2005), *Caulobacter crescentus* (Da Silva Neto, Lourenço & Marques, 2013) and *Pseudomonas aeruginosa* (Nelson et al., 2019) among many others. In *P. aeruginosa*, downregulation of the biosynthesis of amino acids such as cysteine and leucine was also reported in response to iron starvation, together with increases in amino acid catabolic genes. This is probably due to the low energetic status of the cell resulting in reduced anabolism and a shift to energetic pathways alternative to the TCA cycle (Nelson et al., 2019). While repression of histidine biosynthesis by iron in *S. flexneri* agrees with this notion, the overexpression of arginine and cysteine biosynthesis may indicate that a complex, amino acid-specific response occurs in different bacteria. Similarly, tryptophan biosynthesis is induced by iron depletion in *Chlamydia trachomatis*, together with a reduction of expression of translation-related proteins (Brinkworth, Wildung & Carabeo, 2018), with the latter effect also observed in the present study.

A set of genes was regulated by both riboflavin and iron. This result is analogous to that reported in *V. cholerae* (Sepúlveda-Cisternas et al., 2018), although with a lesser degree of overlap between the iron and riboflavin regulons. GO terms enrichment analysis shed light into the physiological functions included in each specific subset of genes. As expected, many of these functions are known to involve electron transfer reactions, such as amino acid biosynthesis, ATP biosynthesis and iron uptake and utilization. Four cellular processes were contrariwise regulated by iron and riboflavin: *ATP synthesis coupled electron transport* and the metabolism of the amino acids *arginine, cysteine and ornithine*. Riboflavin having opposite regulatory effects to those of iron may support the idea that extracellular riboflavin induces a decrease of iron as proposed previously in *V. cholerae* (Sepúlveda-Cisternas et al., 2018).

Transcriptomics showed that riboflavin greatly affected the iron regulon. Genes involved in iron internalization and histidine biosynthesis were affected by iron independently of riboflavin, and thus may represent core iron responses whose expression is independent on the presence of riboflavin. However four more biological processes were added to the iron regulon in riboflavin. Of these, *glutamine family amino acid biosynthetic process* and *proton transmembrane transport* seem related to the general response to iron, namely amino acid and energetic metabolism. Nonetheless, the GO terms *enterobacterial common antigen biosynthetic process* and *amide transport* suggest a more diverse physiological response to iron when riboflavin is present. The enterobacterial common antigen has been linked to resistance to harsh environmental conditions although its function has not been fully elucidated (Goździewicz, Łukasiewicz & Ługowski, 2015). Thus is not clear how its expression could relate to iron physiology. The dramatic increase in the number of genes responsive to iron when riboflavin is present in the medium is intriguing. It suggests that the changes induced by riboflavin in *Shigella* predispose to a wider response to iron. Riboflavin producing the opposite regulatory effect of iron in a number of genes may create a lower threshold of response to iron in these other genes. Thus, this effect should be further studied considering transcriptional regulators and other factors regulated by riboflavin that may be involved in this phenotype. These results also suggest an alteration of iron homeostasis in the presence of environmental riboflavin.

Many studies have previously shown the involvement of iron status on the regulation of the expression of RBP genes. Although riboflavin biosynthetic genes were not responsive to iron in plain T media, the genes *ribB* and *ribH*, coding for the dihydroxybutanone phosphate synthase and lumazine synthase involved in riboflavin biosynthesis were upregulated by iron in the presence of riboflavin. Notably, the rest of the genes of the RBP were not regulated by iron. Unlike other species, the RBP genes in *Shigella* are not encoded in a single operon (Gutiérrez-Preciado et al., 2015). Thus, these results are in accordance with a gene-specific regulatory mechanism of iron over riboflavin biosynthesis, as seen in other bacteria (Pajuelo et al., 2016; Sepúlveda-Cisternas et al., 2018).

Iron also drastically changed the set of genes that responded to riboflavin. Of the 178 genes with altered expression in response to riboflavin in the absence of iron, only four were among the set of 100 genes that responded to riboflavin when iron was added. The metabolism of three amino acids: *methionine, arginine* and *tryptophan*, together with *ATP biosynthesis* were downregulated by riboflavin in low iron. Results showed that siderophore uptake, but not other iron uptake-related functions, was induced by riboflavin in the presence of iron. This induction was accompanied by downregulation of ferritin, which is in line with a decay of iron pools in the presence of riboflavin. Also, cumulative results indicate that iron uptake is mainly expressed under iron deficient conditions, and in such conditions is not responding to riboflavin. The radical change in the nature of the set of genes whose expression was affected by riboflavin indicates that iron has a central role in the physiological functions affected by environmental riboflavin in *S. flexneri*.

Overall, a clear regulatory crosstalk between iron and riboflavin was observed in *S. flexneri*. Nonetheless, is important to note that the metabolic significance of this phenomenon in this species is not clear. In other bacteria, this regulatory feedback has been related to the ability of these two cofactors to coordinately mediate metabolic redox reactions. Nonetheless, *Shigella* genomes lack a recognizable riboflavin transport system (Gutiérrez-Preciado et al., 2015). Thus is currently unknown whether *S. flexneri* is able to uptake environmental riboflavin that could potentially fulfill the redox cofactors needs. Previously, a *S. flexneri* mutant in the *ribE* gene coding for the riboflavin synthase, and hence most likely impaired in endogenous riboflavin biosynthesis, was shown to be viable in rich media (Way et al., 1999). This could suggest that riboflavin is able to enter *Shigella*, whether by a heterologous transport system or through a novel, unidentified riboflavin uptake system. In either case, the presence of extracellular riboflavin has been previously reported to induce physiological changes in *Shigella* species (Deldar & Yakhchali, 2011) and certainly, riboflavin can induce transcriptional changes in a manner independent to its internalization in *V. cholerae* (Sepúlveda-Cisternas et al., 2018). Alternatively, the regulatory effects of riboflavin could be indirectly achieved by altering the oxidation status of extracellular iron. In *Shewanella* and *Listeria*, an extracellular electron transfer process uses flavins as electron shuttles for environmental iron reduction during mineral-respiration (Marsili et al., 2008; Light et al., 2018). Although such systems employ dedicated electron-channeling protein complexes with no orthologs in *Shigella*, this is still a possibility as flavin-mediated reduction of iron proceeds through unknown mechanisms in other bacteria (Crossley et al., 2007). Thus, substantial research is needed to further clarify the biological significance of the regulatory feedback of iron and riboflavin in *S. flexneri*.

## Conclusions

In this study, we applied a transcriptomics approach to evaluate the global interrelations in gene regulation exerted by the presence of riboflavin and iron. Riboflavin and iron seem to inversely affect the expression of a common set of biological functions in *S. flexneri*, while each one additionally regulates a specific subgroup of genes. Also, riboflavin and iron exert marked effects on the regulons of each other. While the presence of riboflavin highly increases the response to iron, iron deeply changes the nature of the riboflavin regulon. In most of the subsets of genes depicted in this analysis, specific GO terms were found to be enriched. This suggests that distinct biological functions may be clustered to respond to different combinations of iron and riboflavin in niches. In general, iron uptake and usage, electron transport chain and amino acids metabolism were the functions more often affected by these two micronutrients. The results of this study provide an outline for the complex relationship between iron and riboflavin in the enteropathogen *S. flexneri* and could prompt systematics studies in other organisms to gain knowledge into this potentially ubiquitous physiological outcome. Moreover, further work is required to identify the mechanisms and molecular players involved in this regulatory crosstalk.

##  Supplemental Information

10.7717/peerj.9553/supp-1 Supplemental Information 1 Quality control results for the RNA samples used for RNAseq RNA quantity and quality were assessed in a 2100 Bioanalyzer. **RIN**: RNA integrity number.

10.7717/peerj.9553/supp-2 Supplemental Information 2 Lists of genes regulated in each transcriptomic comparison

10.7717/peerj.9553/supp-3 Supplemental Information 3 Heatmap of genes responding to both iron and riboflavin An expression analysis of common genes between Comparison *α* (A) and Comparison *β* (B) was performed using heat maps. The excel file containing the genes ID and the value of expression level was uploaded to the web server heatmapper.ca (Babicki, S. et al., Heatmapper: web-enabled heat mapping for all. Nucleic Acids Res. 2016. doi:10.1093/nar/gkw419). The clustering method used was Average Linkage and the distance measurement method used was Euclidean. The scale was adjusted to the values obtained from the transcriptomics data. Red color indicates maximal repression while dark green color indicates the maximum activation of the expression, as indicated by the bar. The name of the genes is indicated to the right in each row.

## Additional Information and Declarations

### Data Availability

The following information was supplied regarding data availability: The transcriptomics paired reads are available at NCBI: PRJNA573757.
