# Supplementary material for: Interdependency of regulatory effects of iron and riboflavin in the foodborne pathogen Shigella flexneri determined by integral transcriptomics
Source: PeerJ. 2020 Sep 15;8:e9553. doi: 10.7717/peerj.9553 (PMC7500357; doi:10.7717/peerj.9553)
Supplement: Supplemental Information 1 — RNA quantity and quality were assessed in a 2100 Bioanalyzer. RIN: RNA integrity number. [file peerj-08-9553-s001.docx]

| Condition | Sample name | Concentration  (ng/µl) | RIN | 23S:16S rRNA  Ratio |
| --- | --- | --- | --- | --- |
| Plain T | ShFe_M_1 | 124.238 | 8.9 | 1 |
|  | ShFe_M_2 | 311.261 | 8.6 | 1 |
|  | ShFe_M_3 | 231.564 | 7.3 | 1 |
| T + Riboflavin | ShFe_M_RF_P1 | 116.687 | 9.3 | 1 |
|  | ShFe_M_RF_P2 | 122.160 | 9.4 | 1 |
|  | ShFe_M_RF_P3 | 96.646 | 9.5 | 1 |
| T + Iron | ShFe_P_1 | 312.449 | 8.8 | 1 |
|  | ShFe_P_2 | 371.050 | 8.3 | 1 |
|  | ShFe_P_3 | 364.487 | 8.7 | 1 |
| T + Riboflavin  + Iron | ShFe_P_RF_P_1 | 271.403 | 7.7 | 0.7 |
|  | ShFe_P_RF_P_2 | 385.081 | 8.8 | 1 |
|  | ShFe_P_RF_P_3 | 426.238 | 8.9 | 1.1 |
