# Supplementary figures and images for: Interdependency of regulatory effects of iron and riboflavin in the foodborne pathogen Shigella flexneri determined by integral transcriptomics
Source: PeerJ. 2020 Sep 15;8:e9553. doi: 10.7717/peerj.9553 (PMC7500357; doi:10.7717/peerj.9553)

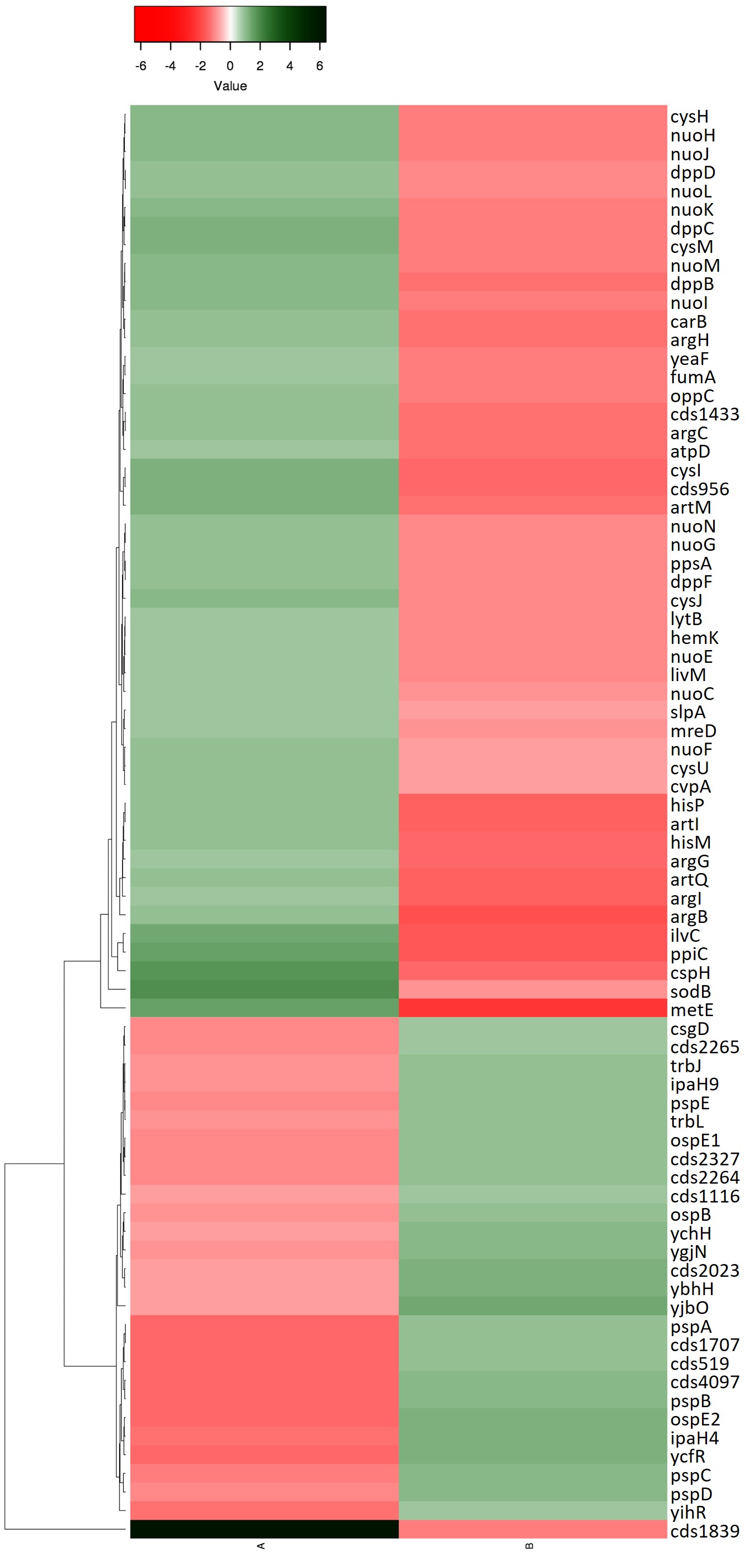

Supplement: Supplemental Information 3 — An expression analysis of common genes between Comparison α (A) and Comparison β (B) was performed using heat maps. The excel file containing the genes ID and the value of expression level was uploaded to the web server heatmapper.ca (Babicki, S. et al., Heatmapper: web-enabled heat mapping for all. Nucleic Acids Res. 2016. doi:10.1093/nar/gkw419). The clustering method used was Average Linkage and the distance measurement method used was Euclidean. The scale was adjusted to the values obtained from the transcriptomics data. Red color indicates maximal repression while dark green color indicates the maximum activation of the expression, as indicated by the bar. The name of the genes is indicated to the right in each row. [file peerj-08-9553-s003.jpg]
